# METTL3‐mediated maturation of miR‐589‐5p promotes the malignant development of liver cancer

**DOI:** 10.1111/jcmm.16845

**Published:** 2022-03-29

**Authors:** Jie Liu, Kai Jiang

**Affiliations:** ^1^ Hepatobiliary and Pancreatic Surgery Department Zhejiang Provincial People’s Hospital People’s Hospital of Hangzhou Medical College Hangzhou China

**Keywords:** liver cancer, m6A methylation, METTL3, miR‐589‐5p, pri‐miR‐589

## Abstract

MiR‐589‐5p could promote liver cancer, but the specific mechanisms are largely unknown. This study examined the role and mechanisms of miR‐589‐5p in liver cancer. The expressions of miR‐589‐5p, METTL3 and m6A in liver cancers were determined by RT‐qPCR. The relationship between miR‐589‐5p and METTL3‐mediated m6A methylation was examined by m6A RNA immunoprecipitation. After transfection, the viability, migration, invasion and expressions of METTL3 and miR‐589‐5p in liver cancer cells were detected by CCK‐8, wound‐healing, transwell and RT‐qPCR. After the xenograft tumour was established in mice, the tumour volume was determined and the expressions of METTL3, miR‐589‐5p, MMP‐2, TIMP‐2, E‐cadherin, N‐cadherin and Vimentin in tumour tissue were detected by RT‐qPCR and Western blotting. *In vitro* study showed that miR‐589‐5p and METTL3 were highly expressed in liver cancer. METTL3 was positively correlated with miR‐589‐5p. METTL3 up‐regulated the expression of miR‐589‐5p and promoted the maturation of miR‐589‐5p. Overexpressed miR‐589‐5p and METTL3 promoted the viability, migration and invasion of liver cancer cells, while the effects of silencing miR‐589‐5p and METTL3 on the cells were the opposite. The effects of METTL3 overexpression and silencing were reversed by miR‐589‐5p inhibitor and mimic, respectively. *In vivo* study showed that METLL3 silencing inhibited the growth of xenograft tumour and the expressions of METTL3, MMP‐2, N‐cadherin and Vimentin, promoted the expressions of TIMP‐2 and E‐cadherin, while miR‐589‐5p mimic caused the opposite results and further reversed the effects of METLL3 silencing. In summary, this study found that METTL3‐mediated maturation of miR‐589‐5p promoted the malignant development of liver cancer.

## 1 INTRODUCTION

Liver cancer, also known as liver hepatocellular carcinoma (LIHC), is one of the most common malignant tumours in humans, and its mortality ranks the third in deaths among all cancers.^1^, ^2^ In clinical practice, liver cancer is mainly treated by surgery, liver transplantation, radiotherapy, chemotherapy and biotherapy.^3^ However, due to a high degree of malignancy, rapid progression, recurrence and metastasis of liver cancer, treatment effectiveness and overall survival of liver cancer patients are still unsatisfactory.^4^ Therefore, it is of great significance to develop novel methods for treating liver cancer.

MicroRNAs (miRNAs) are small non‐coding RNAs that play crucial roles in several physiological and pathological processes in different diseases and cancers, including in liver cancer.^5^, ^6^ For instance, serum miR‐885‐5p in liver cancer patients is a marker for prediction and prognosis of advanced liver cancer^7^; miR‐155‐5p enhances the sensitivity of liver cancer cells to Adriamycin through modulating autophagy and can function as a target to treat liver cancer^8^; miR‐328‐3p inhibits the malignant biological activities of liver cancer cells through regulating ERMP1 and may be a novel marker for liver cancer prognosis.^9^ MiR‐589‐5p is a less studied miRNA, but also has a regulatory effect on liver cancer.^10^ MiR‐589‐5p is highly expressed in liver cancer tissue and facilitates the growth of liver cancer cells.^10^ A study reported that miR‐589‐5p could enhance the maintenance of stemness and chemoresistance of liver cancer.^11^ However, the regulatory mechanisms involved in the effect of miR‐589‐5p in liver cancer are still largely unknown.

N6‐methyladenosine (m6A) RNA methylation, which is a dynamic and reversible modification, is widely discovered in many coding and non‐coding RNAs.^12^ m6A methylation mediates over 80% of RNA methylation and is considered to be a common post‐transcriptional modification of messenger RNA in eukaryotes, and it is also a common post‐transcriptional modification in non‐coding RNAs including in miRNAs.^13^, ^14^ m6A methylation is involved in various aspects of RNA metabolism, such as the regulation of mRNA stability, splicing, transport, localization and translation, RNA‐protein interactions.^15^ m6A on pri‐miRNAs can stimulate miRNA maturation.^16^ m6A participates in various biological processes, including embryogenesis, cancer formation, DNA damage response and spermatogenesis.^15^, ^17^, ^18^, ^19^, ^20^ m6A RNA methylation is conducted by its writers, erasers and readers to add, remove or recognize m6A, respectively. YTHDC1, YTHDC2, YTHDF1, YTHDF2 and YTHDF3 are identified as readers, and readers are proteins, which recognize m6A sites and perform multiple functions in nucleus or cytoplasm.^21^, ^22^ The functional interaction between m6A ‘writers’ and ‘erasers’ determines the dynamic and reversible regulation of m6A modification.^23^ METTL3 is a m6A writer and is identified as the major methyltransferase in the methylation to regulate the development of different types of cancers by modulating the maturation of miRNAs.^12^, ^24^, ^25^, ^26^ For example, METTL3 can modulate the maturation of miR‐126‐5p to promote the progression of ovarian cancer,^27^ and METTL3 enhances the metastasis of colorectal cancer by promoting the maturation of primary miR‐1246 (pri‐miR‐1246).^28^ However, whether the effect of miR‐589‐5p on liver cancer was associated with the METTL3‐mediated m6A methylation has not been researched.

In the present research, we performed *in vitro* and *in vivo* experiments to examine whether the effect and mechanism of miR‐589‐5p on liver cancer was associated with the METTL3‐mediated m6A methylation.

## 2 METHODS

### 2.1 Ethics statement

The collection and use of clinical tissues was approved by the Ethics Committee of Zhejiang Provincial People's Hospital (Z2020042309G), and informed consent was provided by all the patients whose tissues were used in this research. Animal trial in this study was approved by the Committee of Experimental Animals of Zhejiang Provincial People's Hospital (Z2020101006G).

### 2.2 Bioinformatics analysis

The expression of miR‐589‐5p in LIHC was predicted on StarBase (http://starbase.sysu.edu.cn) based on The Cancer Genome Atlas (TCGA) database. The correlation between miR‐589‐5p and METTL3 was analysed on StarBase. The expression of METTL3 in LIHC, diffuse large B‐cell lymphoma (DLBC), cholangiocarcinoma (CHOL), thymoma (THYM) and the survival of patients with LIHC, kidney chromophobe (KICH) and adrenocortical carcinoma (ACC) were analysed by GEPIA2 (http://gepia2.cancer‐pku.cn/#index).

### 2.3 Clinical samples

Liver cancer tissues and adjacent tissues were collected from 70 liver cancer patients who received operation treatment in Zhejiang Provincial People's Hospital. The clinicopathological characteristics (age, sex, hepatitis B virus (HBV), serum alpha‐fetoprotein (AFP) level, tumour size, number of tumour nodules, cirrhosis, venous infiltration, Edmondson‐Steiner grading and tumour‐node‐metastasis (TNM), tumour stage of all the patients with highly expressed or low‐expressed miR‐589‐5p and METTL3) were recorded (Tables 1 and 2).

**TABLE 1:** The clinicopathological characteristics of all patients with high or low expression of miR‐589‐5p

Characteristics	miR−589‐5p		*p*
	Low (*n* = 35)	High (*n* = 35)	
Age (years)			
<50	16	17	0.811
≥50	19	18	
Sex			
Male	25	24	0.794
Female	10	11	
HBV			
Absent	23	20	0.461
Present	12	15	
Serum AFP level (ng/ml)			
<20	12	16	0.329
≥20	23	19	
Tumour size (cm)			
<5	19	10	0.029
≥5	16	25	
No. of tumour nodules			
1	26	24	0.597
≥2	9	11	
Cirrhosis			
Absent	16	12	0.329
Present	19	23	
Venous infiltration			
Absent	22	18	0.334
Present	13	17	
Edmondson‐Steiner grading			
Ⅰ+Ⅱ	20	10	0.016
Ⅲ+Ⅳ	15	25	
TNM tumour stage			
Ⅰ+Ⅱ	21	9	0.004
Ⅲ+Ⅳ	14	26	

**TABLE 2:** The clinicopathological characteristics of all patients with high or low expression of METTL3

Characteristics	METTL3		*P*
	Low (*n* = 35)	High (*n* = 35)	
Age (years)			
<50	17	15	0.631
≥50	18	20	
Sex			
Male	23	18	0.225
Female	12	17	
HBV			
Absent	20	21	0.808
Present	15	14	
Serum AFP level (ng/ml)			
<20	16	20	0.339
≥20	19	15	
Tumour size (cm)			
<5	20	9	0.008
≥5	15	26	
No. of tumour nodules			
1	23	25	0.607
≥2	12	10	
Cirrhosis			
Absent	18	13	0.229
Present	17	22	
Venous infiltration			
Absent	22	19	0.467
Present	13	16	
Edmondson‐Steiner grading			
Ⅰ+Ⅱ	19	10	0.029
Ⅲ+Ⅳ	16	25	
TNM tumour stage			
Ⅰ+Ⅱ	20	8	0.003
Ⅲ+Ⅳ	15	27	

### 2.4 Cell culture

Human normal liver cell line THLE‐2 (CRL‐2706) and human liver cancer cell lines including SNU‐182 (CRL‐2235), SNU‐387 (CRL‐2237), SNU‐423 (CRL‐2238), PLC/PRF/5 (CRL‐8024), Hep3B (HB‐8064) and SK‐Hep1 (HTB‐52) were purchased from ATCC. Human embryonic kidney cell line HEK293T (CL‐0005) was purchased from Procell. All the cells were grown in RPMI 1640 medium (12633012, Gibco) containing 10% FBS (10437010, Gibco) and 1% Penicillin‐Streptomycin (15140163, Gibco) and cultured in a 37°C environment with 5% CO_2_.

### 2.5 RNA m6A quantification

The experiment was performed using the m6A RNA Methylation Assay Kit (ab185912) according to a previous publication.^28^ In brief, three pairs of clinical samples were randomly chosen, and the total RNA in the tissue was isolated by TRIzol (15596018, Invitrogen). After the concentration of the RNA was determined by the Ultra‐micro UV‐Visible Spectrophotometer (NanoDrop, Thermo Scientific), 300 ng RNA was added into the detection well, which was pre‐added with 80 μl binding buffer. After incubation at 37°C for 90 min, the detection well was washed with 150 μl 1 × wash buffer three times. Then, 50 μl capture antibody was added into the well and incubated for 60 min at room temperature, followed by washing with 150 μl 1 × wash buffer for three times. Then, 50 μl detection antibody was added into the well and incubated for 30 min, followed by washing with 150 μl 1 × wash buffer for four times. Then, 50 μl enhancer solution was added into the well and incubated for 30 min, followed by washing with 150 μl 1 × wash buffer for five times. After that, the well was added with 150 μl developer solution and incubated for 5 min. After the well was added with 100 μl stop solution, the absorbance of the well was detected under an Imark microplate reader (Bio‐Rad) under a wavelength of 450 nm.

### 2.6 Mutagenesis assay

This experiment was conducted following a previous publication.^28^ In brief, after the m6A point mutations in pri‐miR‐589 were achieved using the QuikChange Lightning Multi Site‐Directed Mutagenesis Kit (210513; Agilent), the mutant m6A in pri‐miR‐589 (MUT‐pri‐miR‐589) and the wide type pri‐miR‐589 (WT‐pri‐miR‐589) plasmids were constructed. The plasmids were then used to transfect the HEK293T cells.

### 2.7 Cell transfection

MiR‐589‐5p mimic (M; 5′‐UGAGAACCACGUCUGCUCUGAG‐3′), mimic control (MC; 5′‐UCGCAUGAUGUUACCGGAGCAC‐3′), miR‐589‐5p inhibitor (I; 5′‐CUCAGAGCAGACGUGGUUCUCA‐3′), inhibitor control (IC; 5′‐AUAGCCUACGCUGACUGGUCAG‐3′), METTL3 siRNA1 (Sense: AGGCAGCTCATCTGTGTCCT, Anti‐sense: 5′‐UAGUACGGGUAUGUUGAGCTT‐3′), METTL3 siRNA2 (Sense: 5′‐GGUUGGUGUCAAAGGAAAUTT‐3′, Anti‐sense: 5′‐ AUUUCCUUUGACACCAACCTT‐3′), siRNA negative control (siNC; 5′‐GCCTTATTTCTATCTTACGTT‐3′), METTL3 overexpression plasmids and negative control for overexpression (NC) were synthesized by GenePharma. Before transfection, Hep3B, SK‐Hep1 and HEK293T cells were grown in a 6‐well plate, respectively. After the cell confluence became 80%, the siRNA, mimic, inhibitor, plasmids, MUT‐pri‐miR‐589 or WT‐pri‐miR‐589 were respectively transfected into the cells by transfection reagent Lipofectamine 3000 (L3000015, Invitrogen) for 48 h. Finally, the cells were harvested for later use.

### 2.8 m6A RNA immunoprecipitation (MeRIP) assay

This experiment was conducted using the Magna MeRIP m6A Kit (17–10499, Merck) following a previous publication.^28^ In brief, transfected HEK293T cells were collected and lysed by the RIP lysis buffer. 3 μg of m6A antibody (ab208577, Abcam) was conjugated to protein A/G magnetic beads at 4°C for 18 h. Then, the antibody‐conjugated beads were incubated with the antibody in IP buffer with RNase inhibitor and protease inhibitor. The interacting RNAs were isolated and detected by qRT‐PCR.

### 2.9 CCK‐8 assay

After transfection, 1 × 10^3^ Hep3B and SK‐Hep1 cells were added into a 96‐well plate to grow for 24 h, 48 h and 72 h. Then, 10 μl CCK‐8 solution (ab228554, Abcam) was added into each well to incubate the cells for 4 h. Finally, the absorbance under 450 nm was read using a microplate reader.

### 2.10 Wound‐healing assay

After the transfection, 1 × 10^5^ Hep3B and SK‐Hep1 cells were added into a 6‐well plate to the confluence of about 100%. Then, a straight wound was created in each well with a 20‐μl pipette tip. After that, the previous medium was replaced with an FBS‐free medium and incubated for 24 h. The image of each wound at 0 and 24 h was observed under a DMLA optical microscope and analysed by Image J 1.8.0 software.

### 2.11 Transwell assay

Transwell chambers with 8‐μm pore (3422, Corning) were pre‐coated with Matrigel (354234, Corning) and inserted into a 24‐well plate. Then, 1 × 10^5^ transfected Hep3B and SK‐Hep1 cells in 200 μl FBS‐free medium were added into the chambers. 650 μl medium with 10% FBS was added into the 24‐well plate for cell growth for 24 h. Cells invading the lower chambers were stained with purple crystal (C0121, Beyotime, Shanghai, China) for 15 min after being fixed with fixative (P0099, Beyotime). Finally, the dyed cells were observed using an optical microscope and analysed using Image J 1.8.0 software.

### 2.12 Animal study

Forty female 6‐week‐old BALB/c nude mice weighing 20 ± 2 g were purchased from ALF Biotechnology. The mice were fed in an SPF environment (cycle of 12‐h light and 12‐h dark) with a free diet. All the mice were adaptively fed for 5 days before experiments and randomly divided into the following four groups: the siNC+MC group (*n* = 10), the METTL3 siRNA1+MC group (*n* = 10), siNC+M group (*n* = 10) and the METTL3 siRNA1+M group (*n* = 10). Then, the right forelimb of mice in the siNC+MC group was subcutaneously injected with 100 μl PBS containing 1 × 10^6^ SK‐Hep1 cells co‐transfected with siNC and MC; the right forelimb of mice in the METTL3 siRNA1+MC group was subcutaneously injected with 100 μl PBS containing 1 × 10^6^ SK‐Hep1 cells co‐transfected with METTL3 siRNA1 and MC; the right forelimb of mice in the siNC+M group was subcutaneously injected with 100 μl PBS containing 1 × 10^6^ SK‐Hep1 cells co‐transfected with siNC and M; the right forelimb of mice in the METTL3 siRNA1+M group was subcutaneously injected with 100 μl PBS containing 1 × 10^6^ SK‐Hep1 cells co‐transfected with METTL3 siRNA1 and M. After all the mice were normally fed for 30 days, the mice were anaesthetized with 50 mg/kg sodium pentobarbital (B005; Jiancheng) and further sacrificed by cervical dislocation. Subsequently, the tumour was taken out, photographed, and the volume was measured according to the formula that major diameter (L) × (minor diameter)^2^ (*W* ^2^)/2 mm.^3^ Finally, the tumour was used in Western blotting and RT‐qPCR assays.

### 2.13 Western blotting assay

Total proteins from the mice tumour tissues were isolated by NP‐40 buffer (M052443, MREDA). After the protein concentration was determined using a BCA kit (M052460 and MREDA), 20 μg proteins were separated inside the SDS‐PAGE gels (P0052A; Beyotime) and then transferred to the PVDF membrane (M058646 and MREDA), which was blocked with 5% milk for 2 h. After the membrane was incubated overnight at 4°C with the corresponding primary antibodies at the indicated dilutions (Table S1), they were further incubated with secondary antibodies for 2 h at room temperature. Finally, the membrane was incubated with BeyoECL Plus buffer (P0018M, Beyotime) for 1 min, and the protein signal was detected using the Image Lab 3.0 Software (Bio‐Rad).

### 2.14 RT‐qPCR

The mRNA in the clinical samples, cultured cell line and the mice tumour tissues were extracted using TRIzol (M052222 and MREDA), while the miRNA in the clinical samples, cultured cell line and the mice tumour tissues were isolated using an EasyPure miRNA Kit (ER601‐01, TRANS). Then, the total RNA was synthesized into cDNA using BeyoRT III cDNA synthesis regent (D7178L, Beyotime), and miRNA was synthesized into cDNA using TransScript miRNA First‐Strand cDNA Synthesis SuperMix (AT351‐01, TRANS). Finally, after mixing the cDNA with Supermix (AQ601‐01, TransGen) and gene primers (Table S2), the cDNA was amplified in the QuantStudio6 system (Applied Biosystems) and the gene expressions were quantified using the 2^−ΔΔCt^ method.

### 2.15 Statistical analysis

GraphPad 8.0 software was used to analyse the data in this study. The data were expressed as mean ± SD. The data of the two groups were compared using independent sample *t* test (paired sample *t* test between cancerous tissues and adjacent tissues), and one‐way ANOVA was used for the analysis of variance between multiple groups. *p* < 0.05 was considered as statistically significant.

## 3 RESULTS

### 3.1 MiR‐589‐5p was highly expressed in liver cancer tissues and cells

**FIGURE 1:**
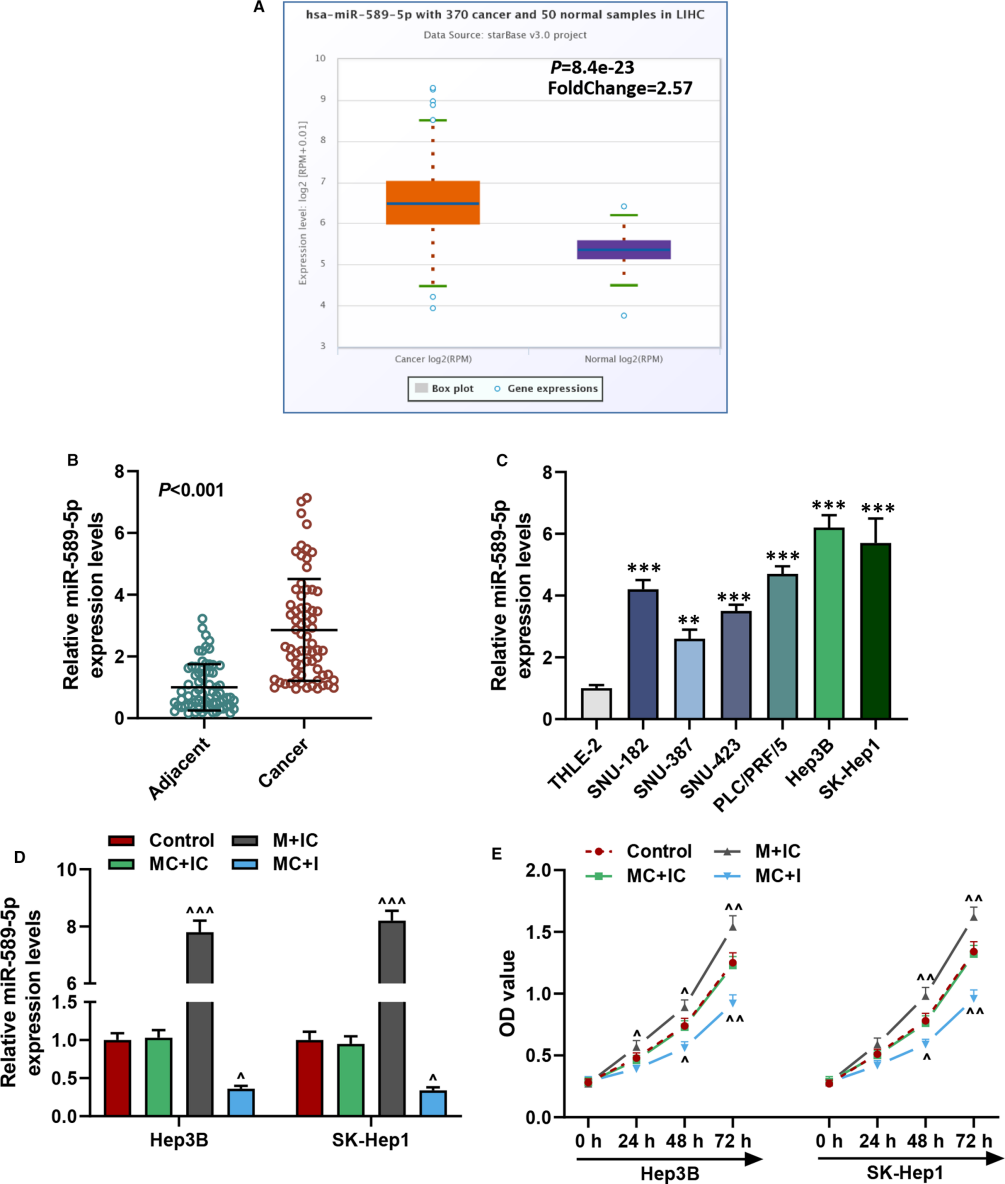
MiR‐589‐5p was highly expressed in liver cancer and regulated the viability of liver cancer cells. (A) The expression of miR‐589‐5p in LIHC was analysed on starBase. (B) The expression of miR‐589‐5p in clinical liver cancer tissues was detected by RT‐qPCR; U6 was an internal control. (C) The expression of miR‐589‐5p in liver cancer cells was detected by RT‐qPCR; U6 was an internal control. (D) The transfection efficiency of miR‐589‐5p mimic and inhibitor in Hep3B and SK‐Hep1 cells was detected by RT‐qPCR, U6 was an internal control. (E) The cell viability of Hep3B and SK‐Hep1 cells after transfection with miR‐589‐5p mimic and inhibitor was detected by CCK‐8 assays. (^**^ *p* < 0.01, ^***^ *p* < 0.001, vs. THLE‐2; ^^^ *p* < 0.01, ^^^^ *p* < 0.01, ^^^^^ *p* < 0.001, vs. MC+IC). (LIHC: liver hepatocellular carcinoma, M; miR‐589‐5p mimic, I: miR‐589‐5p inhibitor, MC: mimic control, IC: inhibitor control)

MiR‐589‐5p was predicted to be highly expressed in LIHC through analysis in starBase (*p* = 8.4e‐23, Figure 1A), and this was further confirmed using clinical liver cancer tissues (*p* < 0.001, Figure 1B) and cultured liver cancer cell lines (*p* < 0.01, Figure 1C) by RT‐qPCR analysis. Furthermore, the clinicopathological characteristics of all patients with high or low expression of miR‐589‐5p were analysed, as exhibited in Table 1; a high level of miR‐589‐5p was associated with the tumour size (*p* = 0.029), Edmondson‐Steiner grading (*p* = 0.016) and TNM tumour stage (*p* = 0.004), indicating that miR‐589‐5p might have a regulatory effect on liver cancer. Given that the level of miR‐589‐5p in Hep3B and SK‐Hep1 was relatively higher than other cancer cells among the 6 liver cancer lines (Figure 1C), the two cells were chosen for later use.

### 3.2 MiR‐589‐5p mimic promoted the viability, migration and invasion of liver cancer cells, but the effects of miR‐589‐5p inhibitor were the opposite

**FIGURE 2:**
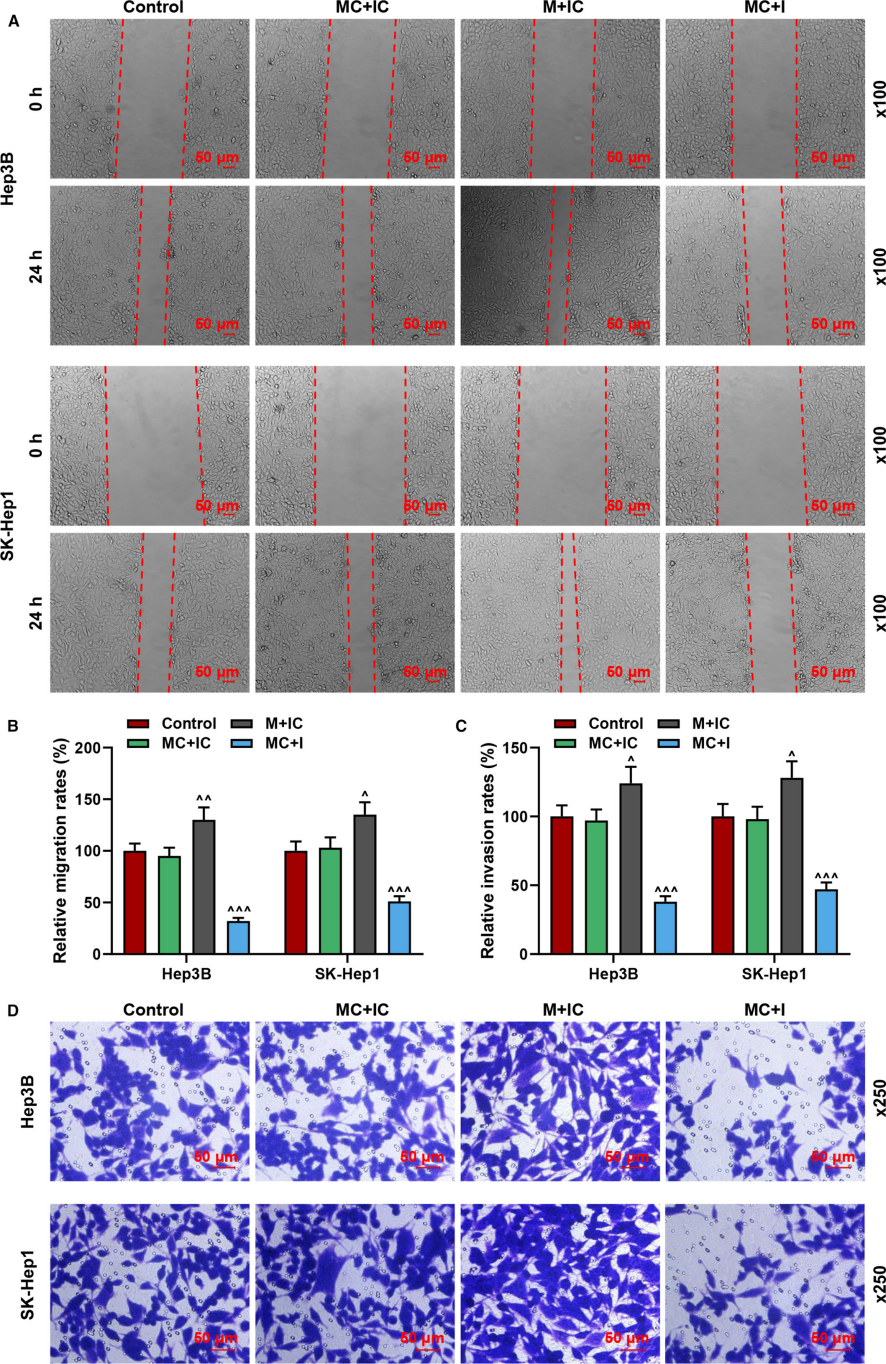
MiR‐589‐5p mimic promoted the migration and invasion of liver cancer cells but the effects of miR‐589‐5p inhibitor were the opposite. (A‐B) The migration of Hep3B and SK‐Hep1 cells after transfection with miR‐589‐5p mimic and inhibitor was detected by wound‐healing assays (Magnification: ×100). (C‐D) The invasion of Hep3B and SK‐Hep1 cells after transfection with miR‐589‐5p mimic and inhibitor was detected by transwell assays (Magnification: ×250). (^^^ *p* < 0.01, ^^^^^ *p* < 0.001, vs. MC+IC). (M; miR‐589‐5p mimic, I: miR‐589‐5p inhibitor, MC: mimic control, IC: inhibitor control)

To investigate the effect of miR‐589‐5p on liver cancer cells, the mimic and inhibitor of miR‐589‐5p were transfected into the Hep3B and SK‐Hep1 cells, as shown in Figure 1D, the expression of miR‐589‐5p was up‐regulated by miR‐589‐5p mimic (*p* < 0.001), but down‐regulated by a miR‐589‐5p inhibitor (*p* < 0.05). After cell transfection, cell biological activities including cell viability and abilities to migrate and invade were evaluated. As shown in Figure 1E, the viability of Hep3B and SK‐Hep1 cells was increased by miR‐589‐5p mimic (*p* < 0.05) but reduced by miR‐589‐5p inhibitor (*p* < 0.05). In addition, miR‐589‐5p further promoted the migration (Figure 2A,B) and invasion (Figure 2C,D) of the cells (*p* < 0.05), while miR‐589‐5p inhibitor inhibited the migration and invasion of Hep3B and SK‐Hep1 cells (*p* < 0.001).

### 3.3 METTL3 was highly expressed in liver cancer, and it was positively correlated to the expression of miR‐589‐5p

**FIGURE 3:**
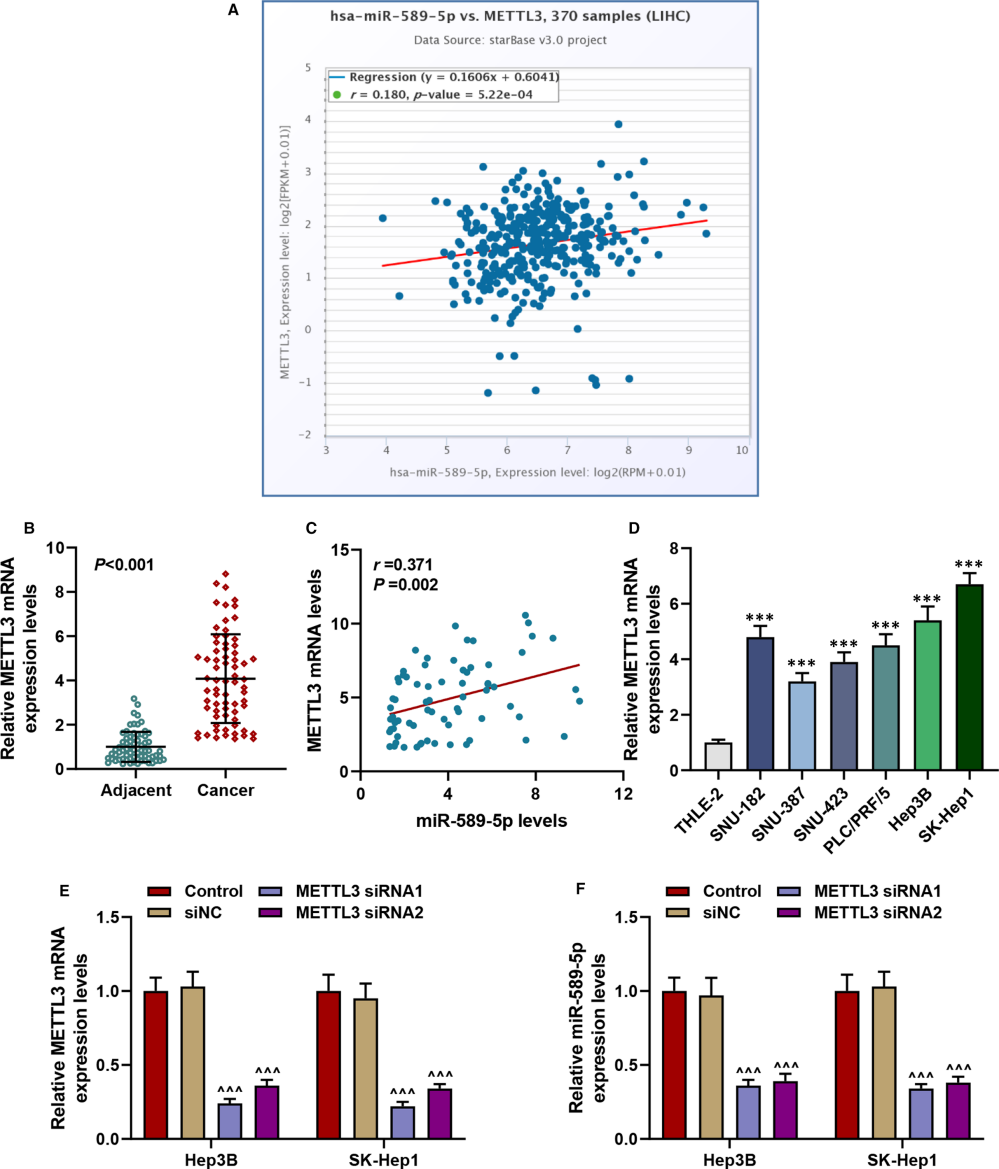
METTL3 was highly expressed in liver cancer and was positively correlated to the expression of miR‐589‐5p. (A) The correlation between METTL3 with miR‐589‐5p was analysed by starBase. (B) The expression of METTL3 in clinical liver cancer samples was analysed by RT‐qPCR, and GAPDH was an internal control. (C) The correlation between METTL3 with miR‐589‐5p in liver cancer samples was analysed by Pearson's correlation analysis. (D) The expression of METTL3 in liver cancer cells was determined by RT‐qPCR, and GAPDH was an internal control. (E) The transfection efficiency of METTL3 siRNA1 and METTL3 siRNA2 in Hep3B and SK‐Hep1 cells was detected by RT‐qPCR, and GAPDH was an internal control. (F) The expression of miR‐589‐5p in Hep3B and SK‐Hep1 cells after silencing METTL3 was detected by RT‐qPCR, and U6 was an internal control. (^***^ *p* < 0.001, vs. THLE‐2; ^^^^^ *p* < 0.001, vs. siNC). (LIHC: liver hepatocellular carcinoma, NC: negative control)

StarBase was used to analyse the correlation between METTL3 and miRNAs, as shown in Figure 3A, METTL3 and miR‐589‐5p were positively correlated. In addition, highly expressed METTL3 in clinical liver cancer samples was further verified (*p* < 0.001, Figure 3B); moreover, the positive correlation of METTL3 and miR‐589‐5p expressions was also confirmed in liver cancer samples (Figure 3C). Meanwhile, as compared with the normal cells, liver cancer cells showed a high level of METTL3 (*p* < 0.001, Figure 3D), indicating that METTL3 had a regulatory effect on liver cancer. To further verify the effect of METTL3 on miR‐589‐5p expression, two siRNAs for METTL3 (METTL3 siRNA1 and METTL3 siRNA2) were transfected into Hep3B and SK‐Hep1 cells for later use. As shown in Figure 3E, the expression of METTL3 in the two cells were both down‐regulated by METTL3 siRNA1 and METTL3 siRNA2 (*p* < 0.001, Figure 3E). In addition, after silencing METTL3 in the two liver cancer cells, the expression of miR‐589‐5p was down‐regulated (*p* < 0.001, Figure 3F).

### 3.4 Highly expressed METTL3 was detected in several cancers and it was correlated with a poor survival of patients

A high level of METTL3 was also detected in DLBC (Figure S1A), CHOL (Figure S1B), THYM (Figure S1C) and LIHC (Figure S1D) through GEPIA2 analysis. Subsequently, the patient overall survival was further analysed, as demonstrated in Figure S1E‐G, the LIHC (Figure S1E), KICH (Figure S1F) and ACC (Figure S1G) patients with a high level of METTL3 developed a poor survival, indicating that METTL3 may be an oncogene for several cancers, including liver cancer. Furthermore, the clinicopathological characteristics of liver cancer patients with a high or low expression of METTL3 were analysed; from Table 2, it could be observed that a high level of METTL3 was associated with the tumour size (*p* = 0.008), Edmondson‐Steiner grading (*p* = 0.029) and TNM tumour stage (*p* = 0.003). Therefore, we then focussed on examining the effect and mechanism of METTL3 in liver cancer.

### 3.5 METTL3 regulated the maturation of miR‐589‐5p by changing the m6A modification status of pri‐miR‐589

**FIGURE 4:**
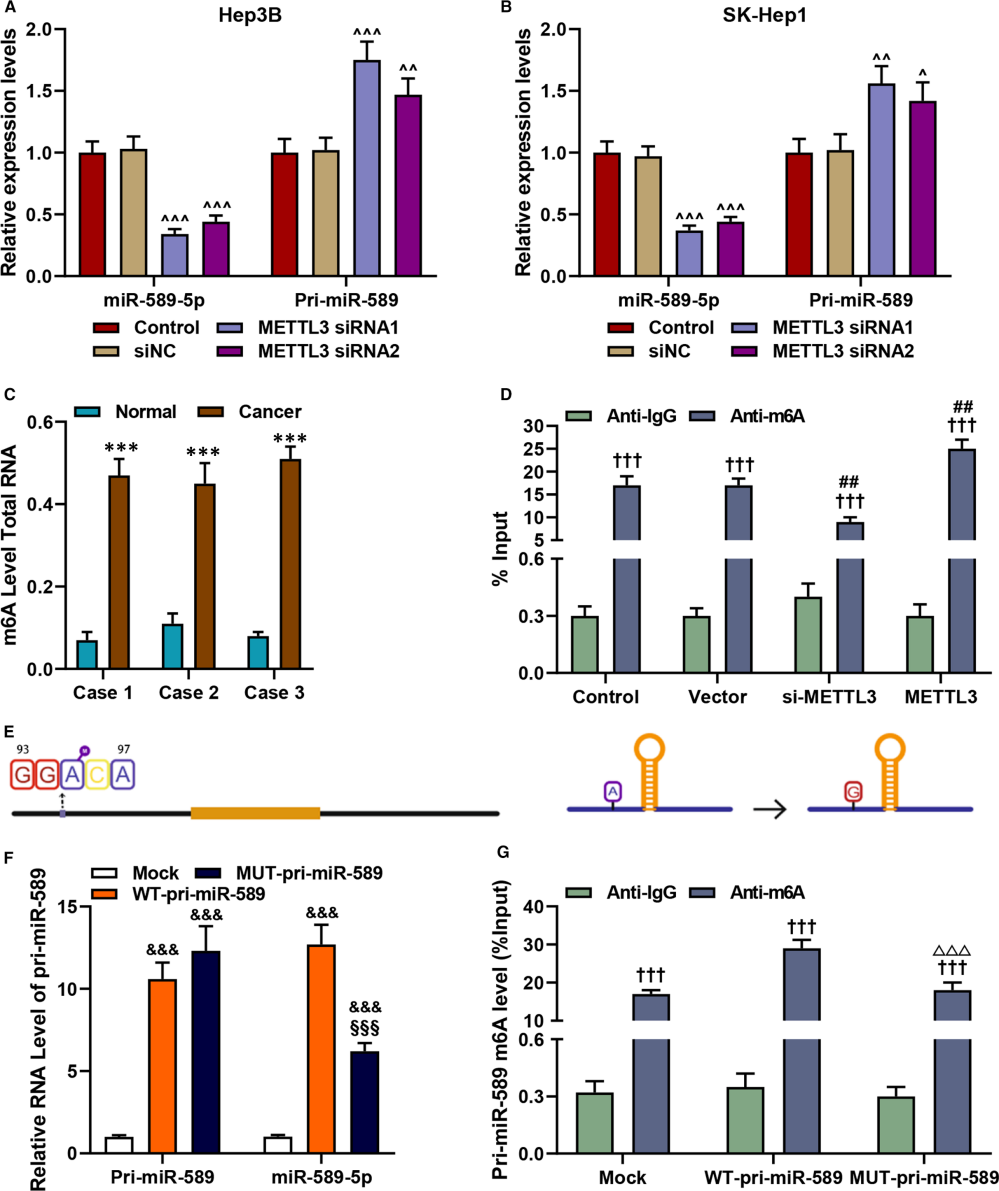
METTL3 regulated the maturation of miR‐589‐5p by changing the m6A modification status of pri‐miR‐589. (A‐B) The expression level of pri‐miR‐589 and miR‐589‐5p in Hep3B (A) and SK‐Hep1 (B) cells after silencing METTL3 was evaluated by RT‐qPCR, and U6 was an internal control. (C) The level of m6A in clinical liver cancer samples was quantified by an m6A RNA Methylation Assay Kit. (D) The m6A modification level in the presence and absence of METTL3 was evaluated by MeRIP assays. (E) GGACA m6A motifs sites were in the upstream of pri‐miR‐589 (Right panel); motif mutation of m6A in pri‐miR‐589 (Left panel). (F) The level of pri‐miR‐589 and miR‐589‐5p after transfected with MUT‐pri‐miR‐589 and WT‐pri‐miR‐589 plasmids in HEK293T cells was detected by RT‐qPCR, and U6 was an internal control. (G) The m6A modification level in the HEK293T cells transfected with MUT‐pri‐miR‐589 and WT‐pri‐miR‐589 plasmids was detected by MeRIP assays. (^^^ *p* < 0.05, ^^^^ *p* < 0.01, ^^^^^ *p* < 0.001, vs. siNC; ^***^ *p* < 0.001, vs. Normal; ^†††^ *p* < 0.001, vs. Anti‐IgG; ^&&&^ *p* < 0.001, vs. Mock; ^##^ *p* < 0.001, vs. Anti‐m6A in Vector group; ^§§§^ *p* < 0.001, vs. WT‐pri‐miR‐589; ^△△△^ *p* < 0.001, vs. Anti‐m6A in WT‐pri‐miR‐589 group). (MeRIP: m6A RNA immunoprecipitation, WT: wide type, MUT: mutant, NC: negative control)

Previous research reported that METTL3 regulates the maturation of certain miRNA.^28^ To determine whether METTL3 regulated the maturation of pri‐miR‐589 to miR‐589‐5p, the expressions of the pri‐miR‐589 and miR‐589‐5p in Hep3B and SK‐Hep1 cells after silencing METTL3 were evaluated. As shown in Figure 4A,B, METTL3 silencing down‐regulated the expression of miR‐589‐5p but up‐regulated the expression of pri‐miR‐589 (*p* < 0.05). METTL3 is associated with the function of m6A modification,^28^, ^29^, ^30^, ^31^ we therefore detected the m6A level in liver cancer samples and discovered that the level of m6A in cancer tissues was significantly up‐regulated as compared with the normal tissues (*p* < 0.001, Figure 4C). Furthermore, the m6A modification was reduced after METTL3 silencing but increased after overexpression METTL3 as compared with the Vector group (*p* < 0.01, Figure 4D). For further research, we obtained the sequences of pri‐miR‐589, pre‐miR‐589‐5p and miR‐589‐5p from University of California Santa Cruz Genomics Institute (UCSC; http://genome.ucsc.edu/), and there were 299 nucleotides of pri‐miR‐589. Then, SRAMP (http://www.cuilab.cn/sramp/) was used to predict the potential modification site of m6A upstream of pri‐miR‐589, and the GGACA m6A motifs were determined to be the m6A deposition site in the upstream of pri‐miR‐589 (Figure 4E, left). To verify the functions of these sites in the m6A modification of pri‐miR‐589, the expression vector containing mutant and wild‐type m6A motif (MUT‐pri‐miR‐589 and WT‐pri‐miR‐589) was constructed. In the MUT‐pri‐miR‐589, guanine replaced the adenine in the m6A motif (Figure 4E, right). Then, the MUT‐pri‐miR‐589 and WT‐pri‐miR‐589 plasmids were transfected into the HEK293T cells (Figure 4F), and we found that the transcript level of pri‐miR‐589 remained unchanged in MUT‐pri‐miR‐589 and WT‐pri‐miR‐589 group, while the transcript level of miR‐589‐5p was down‐regulated in WT‐pri‐miR‐589 group when compared with theWT‐pri‐miR‐589 group (*p* < 0.001). In addition, the down‐regulated m6A level was also discovered in MUT‐pri‐miR‐589 group when compared with the WT‐pri‐miR‐589 group (*p* < 0.001, Figure 4G). These results confirmed that the sites predicted were responsible for m6A modification of pri‐miR‐589, and we further verified that METTL3 regulated the maturation of miR‐589‐5p by changing the m6A modification status of pri‐miR‐589.

### 3.6 METTL3 overexpression promoted the viability, migration and invasion of liver cancer cells by up‐regulating miR‐589‐5p, but the effects of METTL3 silencing were the opposite

**FIGURE 5:**
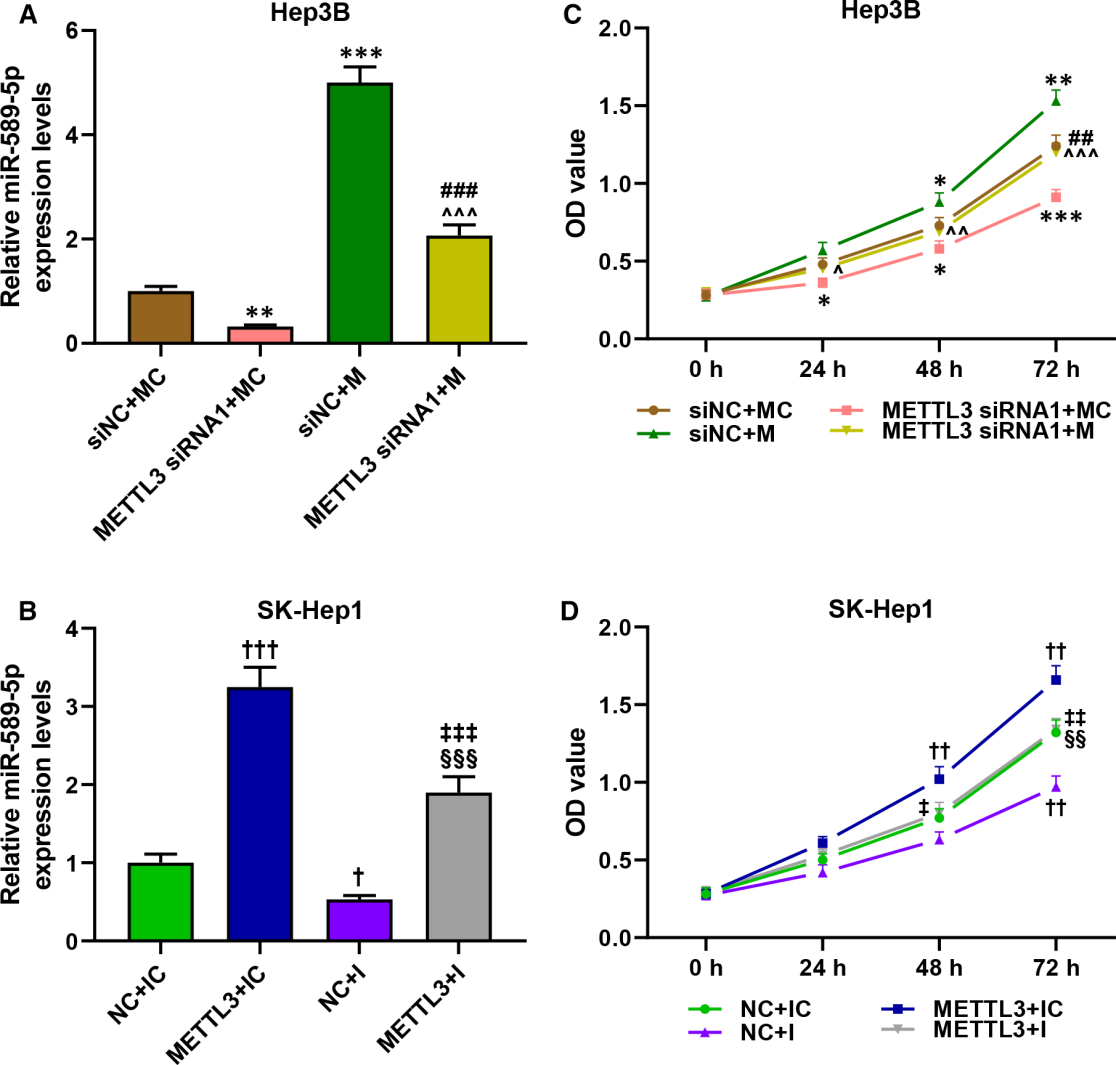
METTL3 regulated the viability of liver cancer cells by regulating miR‐589‐5p. (A‐B) The expression level of miR‐589‐5p in Hep3B (A) and SK‐Hep1 (B) cells after transfection were evaluated by RT‐qPCT, and U6 was an internal control. (C‐D) The viability of Hep3B (C) and SK‐Hep1 (D) cells after transfection were detected by CCK‐8 assays. (^*^ *p* < 0.05, ^**^ *p* < 0.01, ^***^ *p* < 0.001, vs. siNC+MC; ^†^ *p* < 0.05, ^††^ *p* < 0.01, ^†††^ *p* < 0.001, vs. NC+ IC; ^##^ *p* < 0.01, ^###^ *p* < 0.001, vs. METTL3 siRNA1+MC; ^^^ *p* < 0.01, ^^^^ *p* < 0.01,^^^^^ *p* < 0.001, vs. siNC+M; ^‡^ *p* < 0.01, ^‡‡^ *p* < 0.01, ^‡‡‡^ *p* < 0.001, vs. METTL3+IC; ^§§^ *p* < 0.01, ^§§§^ *p* < 0.001, vs. NC+I)

**FIGURE 6:**
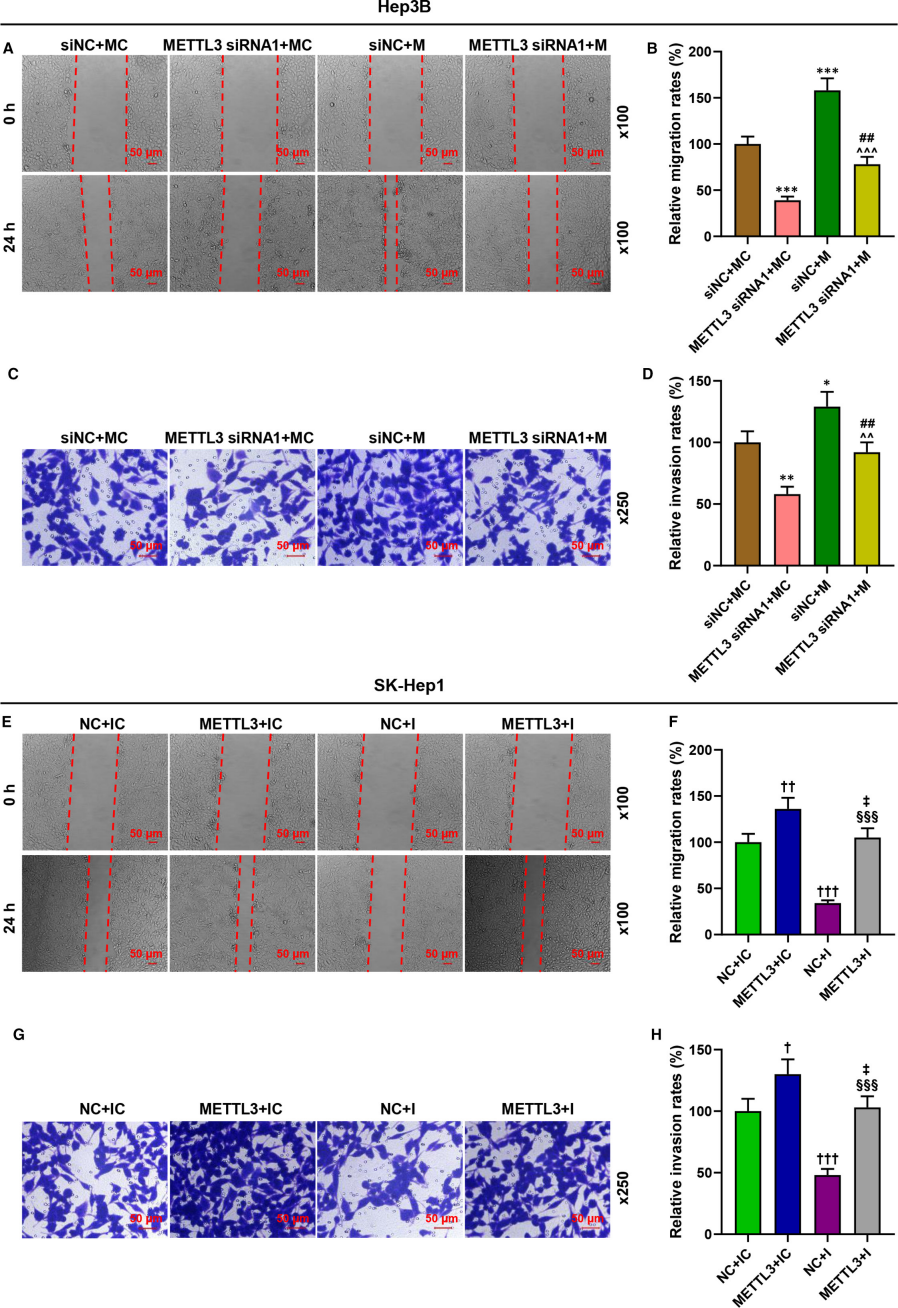
METTL3 regulated the migration and invasion of liver cancer cells by regulating miR‐589‐5p. (A‐B, E‐F) The migration of Hep3B (A‐B) and SK‐Hep1 (E‐F) cells after transfection was evaluated by wound‐healing assays. (C–D, G–H) The invasion of Hep3B (C–D) and SK‐Hep1 (G–H) cells after transfection was detected by transwell assays. (^*^ *p* < 0.05, ^**^ *p* < 0.01, ^***^ *p* < 0.001, vs. siNC+MC; ^†^ *p* < 0.05, ^††^ *p* < 0.01, ^†††^ *p* < 0.001, vs. NC+IC; ^##^ *p* < 0.01, vs. METTL3 siRNA1+MC; ^^^^ *p* < 0.01, ^^^^^ *p* < 0.001, vs. siNC+M; ^‡^ *p* < 0.01, vs. METTL3+IC; ^§§^ *p* < 0.01, ^§§§^ *p* < 0.001, vs. NC+I)

To further verify that the regulatory effect of METTL3 in liver cancer was realized by regulating miR‐589‐5p, the expression of miR‐589‐5p in the Hep3B and SK‐Hep1 cells was detected after the transfection. As exhibited in Figure 5A,B, the expression of miR‐589‐5p was inhibited by METTL3 silencing and miR‐589‐5p inhibitor (*p* < 0.05), while the expression of miR‐589‐5p was promoted by METTL3 overexpression and miR‐589‐5p mimic (*p* < 0.001, Figure 5A,B); moreover, the effect of METTL3 silencing and overexpression on miR‐589‐5p expression was reversed by miR‐589‐5p mimic and inhibitor, respectively. The cell viability (Figure 5C,D), migration (Figure 6A,B,E‐F) and invasion (Figure 6C,D,G,H) of the two cells were reduced by METTL3 silencing and miR‐589‐5p inhibitor (*p* < 0.05), but those cell progresses were increased by METTL3 overexpression and miR‐589‐5p mimic (*p* < 0.05). Also, the effects of METTL3 silencing and overexpression on these processes were reversed by miR‐589‐5p mimic and inhibitor, respectively. These results indicated that METTL3 regulated the viability, migration and invasion of liver cancer cells by regulating miR‐589‐5p.

### 3.7 METTL3 regulated the growth of xenograft tumour by regulating miR‐589‐5p

**FIGURE 7:**
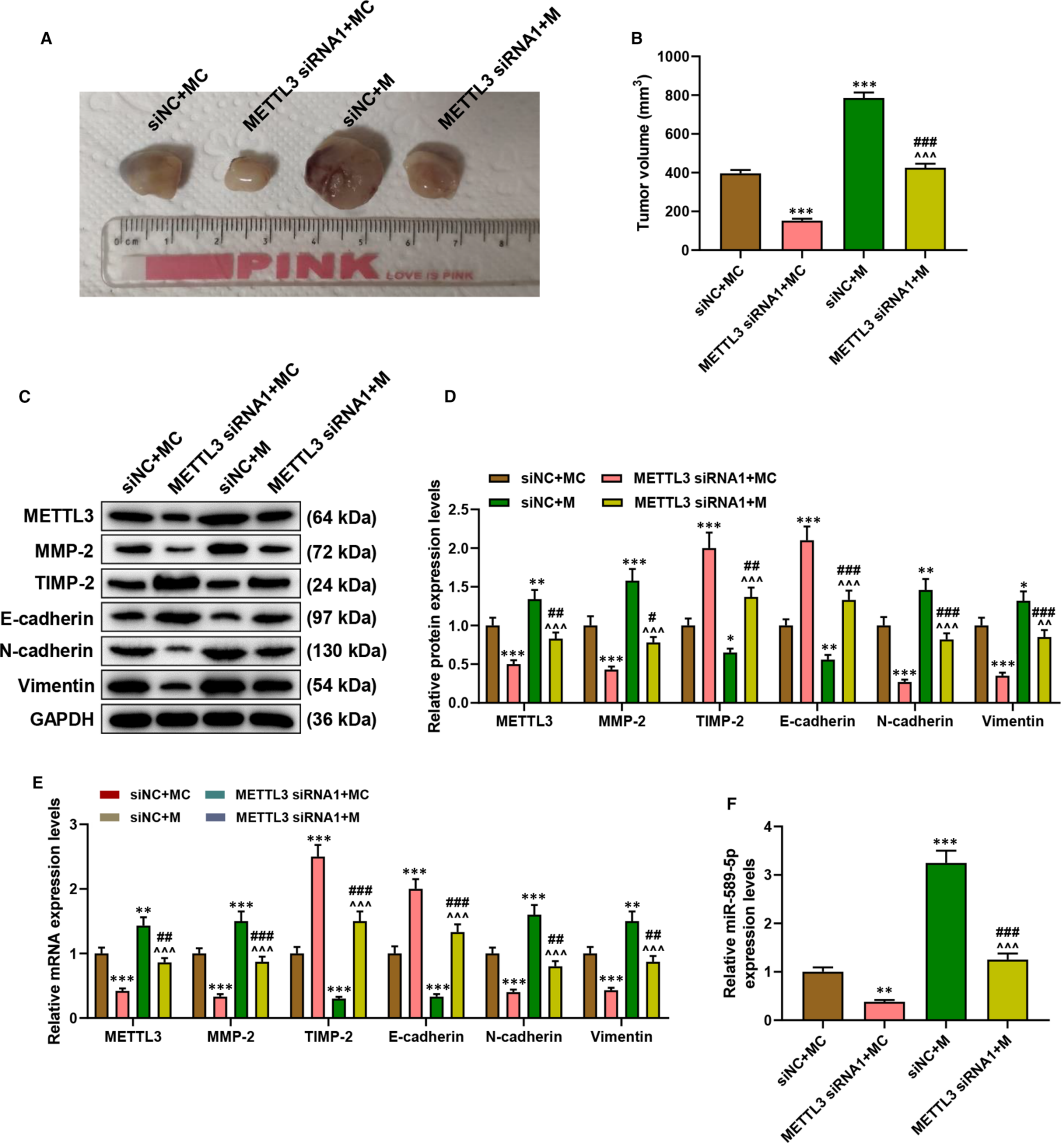
METTL3 regulated the growth of xenograft tumour by regulating miR‐589‐5p. (A) The representative images of xenografted‐tumour. (B) The tumour volume of the xenograft tumour. (C‐E) The expressions of METTL3, MMP‐2, TIMP‐2, E‐cadherin, N‐cadherin and Vimentin in tumour tissue were detected by Western blotting (C‐D) and RT‐qPCR (E), and GAPDH was an internal control. (F) The expression of miR‐589‐5p in the tumour tissue was detected by RT‐qPCR, and U6 was an internal control. (^*^ *p* < 0.05, ^**^ *p* < 0.01, ^***^ *p* < 0.001, vs. siNC+MC; ^#^ *p* < 0.05, ^##^ *p* < 0.01, ^###^ *p* < 0.001, vs. METTL3 siRNA1+MC; ^^^^ *p* < 0.01, ^^^^^ *p* < 0.001, vs. siNC+M)

To confirm the above finding *in vivo*, animal experiments were performed. After the xenograft tumour was collected after growing in mice for 30 days (Figure 7A), we discovered that the tumour volume was inhibited by METTL3 silencing but promoted by miR‐589‐5p mimic as compared with the siNC+MC group (*p* < 0.001, Figure 7B). Also, the effects of METTL3 silencing and miR‐589‐5p mimic on tumour volume were reversed by each other. Then, we detected the expressions of METTL3 and factors related to migration and invasion (Figure 7C‐E) and found that the expressions of METL3, MMP‐2, N‐cadherin and Vimentin were down‐regulated by METTL3 silencing but up‐regulated by miR‐589‐5p mimic (*p* < 0.05), and that the expressions of TIMP‐2 and E‐cadherin were up‐regulated by METTL3 silencing but down‐regulated by miR‐589‐5p mimic (*p* < 0.05). Moreover, the effects of METTL3 silencing and miR‐589‐5p mimic on these factors were reversed by each other. Furthermore, the expression of miR‐589‐5p in the tumour tissue was detected, as shown in Figure 7F, the expression of miR‐589‐5p in the tumour tissue was down‐regulated by METTL3 silencing but up‐regulated by miR‐589‐5p mimic (*p* < 0.01), and the effects of METTL3 silencing and miR‐589‐5p mimic on miR‐589‐5p were reversed by each other. These phenomena suggested that METTL3 regulated the growth of xenograft tumour by regulating miR‐589‐5p.

## 4 DISCUSSION

In this study, we discovered that miR‐589‐5p was highly expressed in liver cancer and promoted the viability, migration and invasion of liver cancer cells and further enhanced the growth of xenograft tumour in nude mice. Moreover, METTL3 was also highly expressed in several cancers, including in liver cancer, and promoted the viability, migration and invasion of liver cancer cells, and METTL3 silencing further inhibited the growth of xenograft tumour in the nude mice. In addition, METTL3 was positively correlated with miR‐589‐5p and enhanced the m6A modification of pri‐miR‐589 to promote the maturation of miR‐589‐5p. Our research revealed that METTL3‐mediated m6A modification promoted the maturation of miR‐589‐5p and the malignant development of liver cancer. Our findings provided new diagnostic and therapeutic markers for liver cancer.

MiR‐589‐5p is a less studied miRNA and has been reported to have a regulatory effect on liver cancer.^10^ Previous research discovered that miR‐589‐5p is promoted in liver cancer.^11^ Consistently, we also proved that miR‐589‐5p was highly expressed in liver cancer tissues and cells. MiR‐589‐5p could promote liver cancer cell viability, migration and invasion.^32^ Similarly, in this study, the viability, migration and invasion of liver cancer cells were promoted by miR‐589‐5p mimic but inhibited by miR‐589‐5p inhibitor, verifying the role of miR‐589‐5p in promoting liver cancer development. However, the mechanism of miR‐589‐5p in affecting liver cancer still needed to be clarified.

m6A methylation mediates over 80% of RNA methylation and is considered to be a common post‐transcriptional modification of messenger RNA in eukaryotes.^13^, ^14^ Current research showed that m6A methylation plays a key role in a variety of pathophysiological processes, such as T cell homeostasis, embryonic thousand cell growth and differentiation and cancer cell growth.^12^, ^13^, ^14^, ^24^ Rich m6A modification has been discovered in ovarian cancer, colorectal cancer, lung cancer and liver cancer.^27^, ^28^, ^33^, ^34^ Similarly, a high level of m6A was also detected in liver cancer in this study. The most recognized underlying mechanism of m6A modification in cancers is that the methylation process of m6A modification is regulated by methyltransferase.^13^ METTL3, as the first reported m6A writer, is a major methyltransferase, and its expression is up‐regulated in different cancers such as gastric cancer, nasopharyngeal carcinoma, cervical cancer and liver cancer.^35^, ^36^, ^37^, ^38^ In the current research, highly expressed METTL3 was further confirmed in DLBC, CHOL, THYM and liver cancer, and the level of METTL3 was determined to be negatively correlated with the overall survival. However, whether m6A modification in liver cancer was mediated by METTL3 remained unknown. Therefore, the MeRIP assay was performed, and for the first time, we further confirmed the association between m6A modification and METTL3 in liver cancer. m6A methylation is also a common post‐transcriptional modification in non‐coding RNAs including in miRNAs. Researches proved that METTL3‐mediated m6A modification promotes the maturation of miRNAs, including miR‐126‐5p, miR‐1246, miR‐221 and miR‐222.^27^, ^28^, ^39^ After we discovered that METTL3 promoted the maturation of miR‐589‐5p, we constructed the mutant and wild‐type m6A motif (in the upstream of pri‐miR‐589) plasmids to further verify whether the effect of METTL3 on the maturation of miR‐589‐5p was associated with the m6A modification of pri‐miR‐589. After performing MeRIP assay, we confirmed that METTL3 promoted the maturation of miR‐589‐5p via the m6A modification of pri‐miR‐589.

To further verify the regulatory effect of METTL3/miR‐589‐5p on liver cancer, the biological activities changes of cancer cells were determined by performing cell experiments, and the growth changes of liver cancer were determined by animal experiments. We found that METTL3 overexpression promoted the viability, migration and invasion of liver cancer cells, and that METTL3 silencing further inhibited the growth of xenografted‐tumour in nude mice but the effects of miR‐589‐5p mimic were the opposite. Moreover, miR‐589‐5p mimic and inhibitor reversed the effects of METTL3 silencing and overexpression on liver cancer, indicating that METTL3/miR‐589‐5p enhanced the malignant development of liver cancer.

Our research, for the first time, verified that METTL3 promoted the maturation of miR‐589‐5p via enhancing m6A modification of pri‐miR‐589 and further enhanced the malignant development of liver cancer. The current findings provide a new diagnostic and therapeutic marker for liver cancer.

## CONFLICT OF INTEREST

The authors declare no conflicts of interest.

## AUTHOR CONTRIBUTIONS

**Jie Liu:** Conceptualization (lead); Data curation (lead); Funding acquisition (equal); Investigation (equal); Methodology (equal); Resources (equal); Writing – original draft (lead); Writing – review and editing (lead). **Kai Jiang:** Data curation (lead); Formal analysis (lead); Methodology (equal); Resources (equal); Supervision (equal).

## Supporting information

Fig S1

Table S1

Table S2
