# Supplementary figures and images for: METTL3‐mediated maturation of miR‐589‐5p promotes the malignant development of liver cancer
Source: J Cell Mol Med. 2022 Mar 29;26(9):2505–19. doi: 10.1111/jcmm.16845 (PMC9077310; doi:10.1111/jcmm.16845)

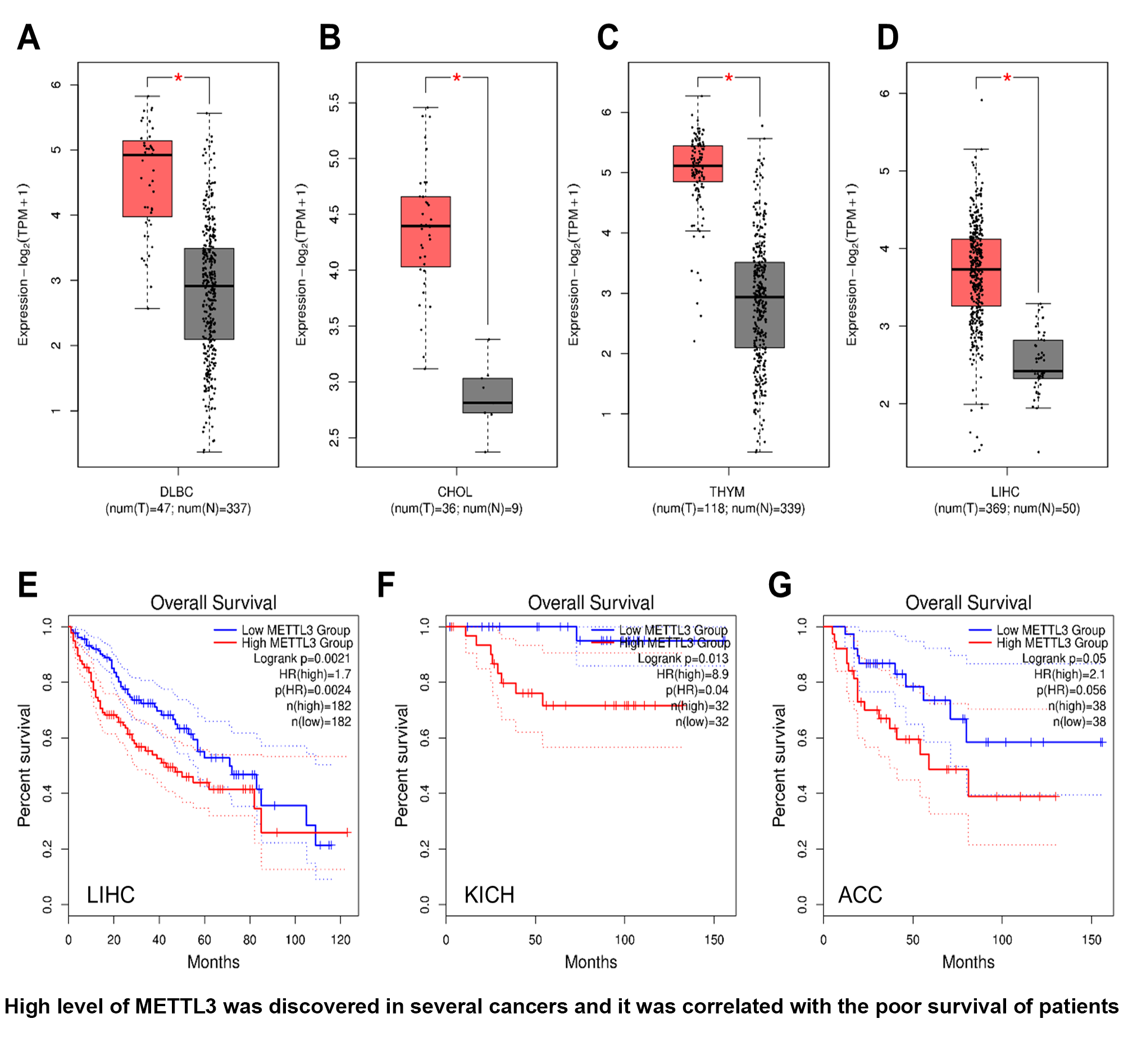

Supplement: Supplementary file 1 — Fig S1 [file JCMM-26-2505-s003.tif]
