# Supplementary material for: METTL3‐mediated maturation of miR‐589‐5p promotes the malignant development of liver cancer
Source: J Cell Mol Med. 2022 Mar 29;26(9):2505–19. doi: 10.1111/jcmm.16845 (PMC9077310; doi:10.1111/jcmm.16845)
Supplement: Supplementary file 2 — Table S1 [file JCMM-26-2505-s001.docx]

Supplementary Table 1 All antibodies information and sources in Western blot in this study

| **Protein name** | **Catalog number** | **Company (country)** | **Molecular weight** | **Dilution ratio** |
| --- | --- | --- | --- | --- |
| METTL3 | ab195352 | Abcam (Cambridge, UK) | 64 kDa | 1/1000 |
| MMP-2 | ab37150 | Abcam (Cambridge, UK) | 72 kDa | 1/2000 |
| N-Cadherin | ab18203 | Abcam (Cambridge, UK) | 130kDa | 1/1500 |
| E-Cadherin | ab40772 | Abcam (Cambridge, UK) | 97 kDa | 1/30000 |
| Vimentin | ab92547 | Abcam (Cambridge, UK) | 54 kDa | 1/4000 |
| GAPDH | ab8245 | Abcam (Cambridge, UK) | 36 kDa | 1/5000 |
| Mouse IgG | ab205719 | Abcam (Cambridge, UK) |  | 1/30000 |
| Rabbit IgG | ab205718 | Abcam (Cambridge, UK) |  | 1/30000 |
