# Supplementary material for: METTL3‐mediated maturation of miR‐589‐5p promotes the malignant development of liver cancer
Source: J Cell Mol Med. 2022 Mar 29;26(9):2505–19. doi: 10.1111/jcmm.16845 (PMC9077310; doi:10.1111/jcmm.16845)
Supplement: Supplementary file 3 — Table S2 [file JCMM-26-2505-s002.docx]

Supplementary Table 2 All RT-qPCR primers in this study

| Target gene | Forward primers, 5’-3’ | Reverse primers, 5’-3’ |
| --- | --- | --- |
| miR-589-5p  Pri-miR-589  METTL3  MMP-2  TIMP-2 | TGAGAACCACGTCTGCTCTG  AACAAATGCCGGTTCCCAGA  AGGCAGCTCATCTGTGTCCT  TACAGGATCATTGGCTACACACC  AAGCGGTCAGTGAGAAGGAAG | TGTCGTGGAGTCGGCAATTG  AGTCGGCAATTGCACTGGAT  GCTTGGCGTGTGGTCTTT  GGTCACATCGCTCCAGACT  GGGGCCGTGTAGATAAACTCTAT |
| E-cadherin | ATTTTTCCCTCGACACCCGAT | TCCCAGGCGTAGACCAAGA |
| N-cadherin | TCAGGCGTCTGTAGAGGCTT | ATGCACATCCTTCGATAAGACTG |
| Vimentin  U6  GAPDH | GACGCCATCAACACCGAGTT  CTCGCTTCGGCAGCACA  ACAACTTTGGTATCGTGGAAGG | CTTTGTCGTTGGTTAGCTGGT  AACGCTTCACGAATTTGCGT  GCCATCACGCCACAGTTTC |
